# Evaluating the implementation of *Nuevo Amanecer-II* in rural community settings using mixed methods and equity frameworks

**DOI:** 10.1186/s13690-023-01207-y

**Published:** 2023-11-09

**Authors:** Jasmine Santoyo-Olsson, Anita L. Stewart, Carmen Ortiz, Helen Palomino, Alma Torres-Nguyen, LaVerne Coleman, Alia Alhomsi, Stephanie Quintero, Jackie Bonilla, Veronica Santana-Ufret, Anna María Nápoles

**Affiliations:** 1 Division of Internal Medicine, University of California San Francisco, San Francisco, CA USA; 2 Institute for Health & Aging, University of California San Francisco, San Francisco, CA USA; 3 Circulo de Vida Cancer Support and Resource Center, San Francisco, CA USA; 4 Cancer Resource Center of the Desert, El Centro, CA USA; 5 Kaweah Delta Health Care District, Visalia, CA USA; 6 WomenCARE/Entre Nosotras, Family Service Agency of the Central Coast, Watsonville, CA USA; 7 Division of Intramural Research, National Institute on Minority Health and Health Disparities, National Institutes of Health, Bethesda, MD USA

**Keywords:** Implementation science, Equity, Breast cancer, Latinas, Psychosocial support, Community-based

## Abstract

**Background:**

The 10-week *Nuevo Amanecer-II* intervention, tested through a randomized controlled trial, reduced anxiety and improved stress management skills among Spanish-speaking Latina breast cancer survivors. This paper describes the implementation and equity evaluation outcomes of the *Nuevo Amanecer-II* intervention delivered in three California rural communities.

**Methods:**

Using implementation and equity frameworks, concurrent convergent mixed methods were applied to evaluate implementation (feasibility, fidelity, acceptability, adoption, appropriateness, and sustainability) and equity (shared power and capacity building) outcomes. Quantitative data were collected using tracking forms, fidelity rating forms, and program evaluation surveys; qualitative data were collected using semi-structured in-depth interviews. Respondents included community-based organization (CBO) administrators, recruiters, compañeras (interventionists), and program participants.

**Results:**

Of 76 women randomized to the intervention, 65 (86%) completed at least 7 of 10 sessions. Participants’ knowledge (85% correct of 7 questions) and skills mastery were high (85% able to correctly perform 14 skills). Mean fidelity ratings across compañeras ranged from 3.8 (modeled skills) to 5.0 (used supportive/caring communication); 1–5 scale. The program was rated as very good/excellent by 90% of participants. Participants and compañeras suggested including family members; compañeras suggested expanding content on managing thoughts and mood and healthy living and having access to participant’s survivorship care plan to tailor breast cancer information. CBOs adopted the program because it aligned with their priority populations and mission. Building on CBOs’ knowledge, resources, and infrastructure, implementation success was due to shared power, learning, responsibility, and co-ownership, resulting in a co-created tailored program for community and organizational contexts. Building intervention capacity prior to implementation, providing funding, and ongoing technical support to CBOs were vital for fidelity and enhancement of recruiter and compañera professional skills. Two of three CBOs created plans for program sustainability beyond the clinical trial; all administrators discussed the need for new funding sources to sustain the program as delivered.

**Conclusions:**

Building on community assets and using equitable participatory research processes were central to the successful implementation of a peer-delivered psychosocial intervention in three rural communities among Spanish-speaking Latinas with breast cancer.

**Supplementary Information:**

The online version contains supplementary material available at 10.1186/s13690-023-01207-y.

**Table:** 

**Text box 1. Contributions to the literature**
• We evaluate implementation of a 10-week peer delivered stress management program for Spanish-speaking Latinas with breast cancer, in three rural California communities, using mixed-methods to comprehensively evaluate the implementation process from multiple perspectives.
• We apply implementation and equity frameworks to evaluate implementation (feasibility, fidelity, acceptability, adoption, appropriateness, sustainability) and equity (shared power, capacity building) outcomes.
• Implementation success was due largely to use of shared responsibility and learning, and co-ownership strategies, resulting in a co-created, co-tailored program for Spanish-speaking Latinas with breast cancer and the community and organizational context.
• We summarize best practices for how investigators can equitably engage community organizations to implement behavioral interventions.

## Background

Psychosocial interventions for women with breast cancer can effectively decrease psychological distress and improve quality of life [1, 2]. These interventions have been designed and tested in a wide variety of populations and settings [3]. They have been implemented within health care, public health, and community-based organization (CBO) settings. They have been delivered by a range of interventionists from health professionals to trained peers or community health workers. To reach non-white or limited English proficient women, interventions have been translated and delivered in the client’s native language(s) [4, 5]. The interventions typically have small but beneficial effects on various psychosocial outcomes (i.e., quality of life, depression, cancer-related distress, and anxiety) [5].

Despite efforts to provide supportive services to women with breast cancer, very few have been offered to rural Latinas with breast cancer [6–8], representing a significant science-to-practice gap. Throughout rural California, Latinos disproportionately live in poverty and medically underserved areas, and fewer have a high school degree/some college, compared to Latinos in urban areas [9–11]. Providing programs in rural areas requires addressing specific barriers such as limited English proficiency (LEP), low literacy, travel distances, lack of transportation, limited insurance coverage, unfamiliarity with the healthcare systems, and problems paying for medical care [12, 13]. Despite the need in rural communities, psychosocial interventions are rarely tested in these low-resourced and challenging settings [14, 15].

To fill this gap, we designed a stress reduction intervention specifically for rural-dwelling Spanish-speaking breast cancer survivors. We partnered with three CBOs in rural settings to translate/adapt our first-generation *Nuevo Amanecer* (*NA*) program into *Nuevo Amanecer-II* (*NA-II*), after demonstrating the efficacy of *NA* among urban Latinas living with newly diagnosed breast cancer [16]. The adaptations made to *NA* for these rural settings are described elsewhere [17]. The effectiveness of *NA-II* was evaluated with a 6-month randomized controlled trial (RCT), and was found to reduce anxiety and improve stress management skills [18]. Briefly, this RCT was conducted in real-world settings with the aim to increase generalizability to women throughout survivorship. Thus, eligibility was open to women regardless of time since diagnosis. Trained, community‐based recruiters conducted in‐person 60‐minute baseline assessments (in Spanish) in the participant’s home or CBO’s office. Randomization to receive the intervention or control group was stratified by recruitment site. Before initiating recruitment, stratum‐specific sequential identification numbers were generated and randomly preassigned in blocks of random sizes. The individual was the unit of randomization with 1:1 allocation to experimental groups. Academic researchers were blind to group assignment. In total 153 primarily monolingual (85%) Spanish-speaking Latina women with non-metastatic breast cancer were randomized. The majority were Mexican immigrants (97%) and most had less than high school education (69%). This paper describes the evaluation of the implementation of *NA-II* within three rural communities using the Proctor implementation Outcomes Framework [19] and the Conceptual Model for Evaluating Equity within the Context of Community-based Participatory Research (CBPR) Partnerships (henceforth, Conceptual Model for Evaluating Equity) [20]. Results can inform future efforts by investigators implementing behavioral interventions in rural communities.

## Methods

### Nuevo Amanecer-II (NA-II) overview

#### Partners and partnership approaches

*NA-II* was a partnership between the University of California San Francisco (UCSF) (academic partner), Círculo de Vida Cancer Support and Resource Center (lead community partner/compañera (interventionist) supervisor), and three CBOs that implemented the program in rural California communities. The lead community partner is a bilingual-bicultural clinical psychologist, Co-Principal Investigator on the study, and Executive Director of a San-Francisco-based CBO providing cancer support services to Latinos. Partnering CBOs serving as implementation sites included: WomenCARE (Watsonville, CA), Kaweah Delta Health Care District (Visalia, CA), and Cancer Resource Center of the Desert (El Centro, CA). Sites are described in detail elsewhere [17].

Implementation of *NA-II* was guided by the Transcreation Framework [21] and CBPR principles (e.g., trust, shared decision-making, equal value placed on scientific and community knowledge) [22]. *NA-II* partners were engaged in all research phases (i.e., program adaption/co-creation, implementation, evaluation, interpretation of results, and dissemination). Monthly partnership meetings included all study staff (UCSF-academic partner, lead community partner staff, and individuals from three CBO implementation sites (administrators, recruiters, and compañeras). The partnership shared responsibility for and ownership of intervention and data collection activities, while emphasizing CBO’s strengths and resources and building capacity [23].

#### Personnel and organizational structure

CBOs received funds for study implementation ($45,000 each) and controlled their budget. Three types of CBO personnel participated in the study: administrators, recruiters, and compañeras (interventionists). One administrator from each CBO was actively involved throughout the study, serving as key decision maker regarding program design and implementation. Input was secured throughout from all study staff. Two CBO staff members or volunteers were identified by each administrator to be recruiters. Recruiters promoted *NA-II* in the community, explained the study, and enrolled eligible women (consent, baseline survey, and randomization into the RCT). Two individuals (Latina breast cancer survivors at least three years post-diagnosis with no recurrence) were identified by each CBO to deliver the intervention (compañeras). Recruiters and compañeras were trained by the academic and lead community partners (Co-Principal Investigators). The lead community partner supervised the compañeras in the field.

#### Nuevo Amanecer-II program

*NA-II* was a 10-week structured program delivered in Spanish by a trained compañera in the woman’s home or alternate site chosen by participants. Structured weekly modules provided training in cognitive-behavioral coping skills to manage stress and emotions and emotional support from the compañera (a culturally similar breast cancer survivor). The program is described in detail elsewhere [17]. Sessions included a deep breathing practice, review of the prior session to reinforce key concepts, review of the new week’s material, hands-on exercises, modeling and coaching by the compañera, role-playing, and a recap of the new material and weekly goals to practice new skills introduced. Women received a program manual and DVD containing stress management and breast cancer informational videos with instructions and a YouTube link. During sessions, compañeras and participants used the manual and pre-loaded tablet with videos to practice skills and review information.

#### Study design and frameworks for evaluating implementation and equity

We used a concurrent convergent mixed-methods design with qualitative and quantitative data collected from multiple perspectives (CBO administrators, recruiters, compañeras, compañera supervisor, and participants). Multiple data types were collected concurrently, analyzed separately, and then integrated and converged to conduct a comprehensive equity-informed implementation process evaluation [24]. The evaluation is guided by the Proctor implementation outcomes framework [19] and the Conceptual Model for Evaluating Equity [20]. Based on these frameworks, evaluation outcomes specified a priori were: implementation (feasibility, fidelity, acceptability, adoption, appropriateness, and sustainability) and equity outcomes (shared power and capacity building). The Proctor framework was selected due to its distinct yet inter-related implementation outcomes [19]. The Conceptual Model for Evaluating Equity was employed to evaluate CBPR partnership equity outcomes outlined by Ward and colleagues [20]. Equity outcomes were included as the literature highlights the challenges of communication, inclusiveness, and community involvement to successful implementation process [25] and successful CBPR [20].

### Respondents

CBO administrators, recruiters, and compañeras were contacted for a semi-structured interview about program implementation. Compañeras completed a structured program tracking form for all intervention group participants (end-users) after each weekly session. The compañera supervisor completed a structured fidelity rating form for observed program sessions. All participants completing the program (including intervention and wait-list control group women who elected to receive the program after the final outcomes survey) were invited to complete a structured program evaluation survey. We randomly selected 10 participants who completed the program evaluation survey for a semi-structured interview about their experiences.

### Data collection

Five types (sources) of program evaluation data were collected. Data were managed using a secure REDCap [26] data system.

#### RCT tracking form

Recruiters used a paper tracking form to document recruitment and retention for each potential participant (name obtained through outreach). The form included name, contact information, study ID, study eligibility questions, and a check list of study enrollment requirements (study consent, baseline survey, and randomization), with places to record dates/times of phone calls/contacts, recruitment disposition (e.g., enrolled, not interested), and reasons why participants did not enroll (e.g., too busy). The academic team entered all RCT tracking forms into the REDCap data system. Similar tracking forms were used for women enrolled in the study that included a check list of 3-month study requirements: 3-month survey, program evaluation survey (if assigned to intervention group) and 6-month survey. The disposition (e.g., completed survey, loss to follow-up) and reasons why participants did not complete the surveys (e.g., too busy, disconnected phone) or reasons that intervention group women discontinued the study at any point (e.g., experiencing serious treatment side effects, traveling) were documented. The academic team used tracking forms to assess retention rates.

#### Fidelity rating form

The compañera supervisor made site visits to CBOs to directly observe intervention sessions (1–2 intervention sessions per compañera). Using structured rating scales (1 = not at all to 5 = all the time), the supervisor rated compliance with six program components (the extent to which they followed the manual for that session, explained concepts in language the participant understood, checked that participant understood the material, modeled the skills, spoke in a supportive/caring way, and provided praise/feedback to participant when practiced the skills) and the extent to which compañeras encouraged participants to practice the seven skills being taught.

#### Program tracking form

Compañeras completed structured program tracking forms after each session and recorded program attendance and logistics, reasons why participants missed a session, and several aspects of program uptake (whether participant completed the assigned goal(s) for that week (yes or no), whether the participant reported difficulty in doing the goal (yes or no) and type of difficulty (open-ended), whether participants were able to answer correctly a few questions about a session’s material (correct or incorrect), and whether they were able to demonstrate skills covered in the prior session (yes or no).

#### Program evaluation survey

A few weeks after completing the program, a structured program evaluation survey was administered by telephone by a bilingual-bicultural research associate to participants who completed at least 7 of 10 sessions. The interview lasted about 10-minutes and women received $10.

#### Semi-structured interviews

After the RCT, all CBO administrators, recruiters, compañeras, and a subsample of participants were invited to semi-structured interviews via telephone to debrief them about their experiences in implementing the program and participating in the study. Interviews with administrators were conducted in English (by informants’ choice) by a trained bilingual-bicultural interviewer and lasted 60-minutes. Interviews with recruiters and compañeras were conducted in Spanish (by informants’ choice) by a trained bilingual-bicultural interviewer and lasted 90-minutes. Administrators, recruiters, and compañeras each received $50. Participant semi-structured interviews were conducted in Spanish via telephone by a trained bilingual-bicultural interviewer; the interview lasted 30-minutes, and each participant received $25.

Semi-structured interviews were audio-recorded and transcribed verbatim in English or Spanish by a professional transcription service. Transcriptions were de-identified and were analyzed in their original language to prevent nuances from getting ‘lost in translation’ [27].

### Implementation outcomes

Implementation outcomes include: feasibility, fidelity, acceptability, adoption, appropriateness, and sustainability [19]. Shared power and capacity building were the equity outcomes of interest because of the importance of communication, inclusiveness, and community involvement to successful implementation and CBPR processes [20, 25]. Table 1 provides an overview of the outcomes with definitions, operationalization (content), respondent, and data source.

**Table 1:** Outcomes, operationalization, respondents, and methods of data collection

Outcome*	Operationalization	Respondent	Data source
**Implementation Outcomes**			
Feasibility	• Extent to which recruitment and retention rates met study goals across three CBOs in rural communities	• Community recruiters • Academic research team	• RCT tracking form
	• Program dose: completing ≥ 7 of 10 sessions	• Compañeras	• Program tracking form
Fidelity	• Participants’ uptake of the program (e.g., completion of weekly goals, ability to demonstrate skills, etc.)	• Compañeras	• Program tracking form
	• Compañeras delivery of the program protocol (e.g., follow manual, model skills, etc.) • Direct observation of the quality of program delivery	• Compañera supervisor	• Fidelity rating form
Acceptability	• Participants’ ratings of overall quality, perceived usefulness, ease of use, preferences for program format, and suggestions for program improvement	• Participants	• Program evaluation survey • Semi-structured interview
	• Compañeras’ perceptions on acceptability of – program format, quality, benefits, ease of use, and suggestions for program improvement	• Compañeras	• Semi-structured interview
Adoption	• Factors affecting CBO administrators’ decision to implement the program (e.g., infrastructure, health priority, existing resources, etc.)	• CBO administrators	• Semi-structured interview
Appropriateness	• Perceptions of fit or practicability of program for CBO or participants	• CBO administrators • Compañeras	• Semi-structured interview
	• Perceptions of fit or practicability of research methods (enrollment procedures – consent, baseline interview, randomization) for community recruiters or participants	• CBO administrators • Community recruiters	• Semi-structured interview
Sustainability	• CBO administrators’ plans to sustain the program within their CBO	• CBO administrators	• Semi-structured interview
**Equity Outcomes**			
Shared power	• Perceived partnership dynamics (e.g., leadership, communication, governance, partnership challenges, etc.) among community partners	• CBO administrators • Community recruiters • Compañeras	• Semi-structured interview
Capacity building	• Perceived capacity building (e.g., knowledge, service/expertise, reputation, etc.) among CBO partners	• CBO administrators • Community recruiters • Compañeras	• Semi-structured interview

*Implementation outcomes per the Proctor implementation outcomes framework [19] and equity outcomes definitions per the Conceptual Model for Evaluating Equity within the Context of CBPR Partnerships [20]

*Feasibility* is defined as the extent to which a program can be successfully used or carried out within a given setting [19]. We focused on the feasibility of recruitment and retention, and dose of the program received. The overall RCT enrollment goal was 150 women across all three sites; thus each organization was responsible for enrolling 50 women. Retention at 6 months was defined as completing the 6-month study survey. The retention goal was 90% at 6 months. Data on recruitment and retention were collected on the RCT tracking form. Program dose was measured by the number of program sessions attended as recorded by compañeras on the program tracking form. Program adherence was defined as having completed at least 7 of 10 sessions.

*Fidelity* is the degree to which a program was implemented as described in the original protocol [19]. For *NA-II*, fidelity was operationalized separately for participants (adherence to program) and compañeras (adherence to program delivery). For participants, fidelity was operationalized in terms of 1) participants’ adherence to and uptake of the program protocol as noted on tracking forms by compañeras. For compañeras, fidelity was operationalized in terms of (1) compañeras’ adherence to the program delivery protocol, and (2) the quality of program delivery, based on supervisor ratings during directly observed sessions.

*Acceptability* reflects participants’ and compañeras’ perceptions of whether the program was agreeable, palatable, or satisfactory [19]. To assess acceptability, we used participants’ program evaluation surveys and semi-structured interviews with participants and compañeras. Using structured response choices, the program evaluation survey assessed participant’s program acceptability, specifically: participants’ format preferences (timing, number of sessions, and delivery format); quality of the program, videos, and compañera skills; perceived usefulness (how much the program helped them cope with breast cancer); ease of use; and suggestions for program improvement. Women rated the usefulness of each session content/topic (i.e., cancer information, survivorship care plan, communicating with doctors, communicating with family members, managing thoughts and mood, managing stress, healthy living, and setting goals). Ease of use was assessed by asking how easy it was to understand the manual, how convenient the program was, and how often they continued to practice the skills learned after completing the program. Participant semi-structured interview questions were parallel to the program evaluation survey but more in-depth. In the compañera semi-structured interview, we asked about their perceptions of program acceptability, usefulness of program content and materials, appropriateness of format and delivery, how the program helped participant’s cope, whether participants understood or had problems understanding content or materials, barriers to successful completion of sessions and how these might be overcome, and suggestions for improvements.

*Adoption* is defined as the intention, initial decision, or action to employ an evidence-based program by CBO administrators as part of the real-world implementation efforts in their settings [19]. We asked about administrators’ initial decisions to implement *NA-II* and its relevance to their site and community needs.

*Appropriateness* reflects perceptions of the fit or practicability of the program and research methods [19]. Appropriateness was assessed through semi-structured interviews with recruiters, compañeras, and CBO administrators. Recruiters were asked about the appropriateness of recruitment and enrollment methods, e.g., outreach, recruitment, consent, baseline interview, randomization, and strategies for reaching more women. Compañeras and administrators were asked about their involvement in tailoring the program for their clients and settings. Administrators were also asked about hiring and supervision of compañeras and recruiters.

*Sustainability* is defined as the extent to which a newly implemented program is maintained or institutionalized within a CBO’s ongoing operations [19]. Sustainability was assessed through semi-structured interviews with administrators asking them about incentives/disincentives to implementing the program (e.g., resources, infrastructure), barriers and facilitators to program implementation at the individual, organization, and community levels, and plans for program sustainability.

### Equity outcomes

*Shared power* reflects the perceptions of individuals engaged in the partnership including leadership, dynamics, communication, decision-making, resources, governance mechanisms, efficiency, and partnership challenges [20, 28]. Related semi-structured interview questions included, “How could the communication between your organization and the research team be improved?”; “How did the research team take into account your organization’s unique needs?”; “What was the leadership style of the research team?” Community recruiters and compañeras were asked parallel questions.

*Capacity building* reflects perceptions of personal growth (e.g., expertise, knowledge gained, personal skills) or how their organization was enhanced (e.g., services, reputation) as a result of the partnership [20, 28]. Related semi-structured interview questions for administrators and compañeras included, “How did the training you received through the *Nuevo Amanecer* program benefit your organization?” and “What were the changes in your community or organization as a result of this study?”

### Data analyses

We first analyzed quantitative and qualitative data separately then converged qualitative and quantitative findings.

Feasibility data from the RCT and compañera program tracking forms were summarized in terms of frequencies and percentages for the recruitment rate (participants enrolled/invited/ineligible), retention (completed 6-month survey), and program dose (completed at least 7 program sessions/assigned to intervention group).

The compañera supervisor’s fidelity rating forms were summarized across compañeras using means and standard deviations. For the seven skills, we report the frequency of how much compañeras encouraged participants to practice skills.

Acceptability outcomes from the structured program evaluation survey were summarized in terms of frequencies and percentages. Semi-structured interviews with participants and compañeras were analyzed using a deductive thematic approach [29] using Dedoose software [30]. The program evaluation survey was used to create a structured program codebook since, as described above, the participant and compañera semi-structured interview questions were parallel to the program evaluation survey but more in-depth. The structured program codebook was replicated in Dedoose to organize and analyze the data. Analysis started with one author (JS-O) coding interview transcripts to ascertain themes and constructs aligned with the structured program codebook. Two coders then independently coded each interview using the structured program codebook then reviewed the themes to determine coding consensus. Themes were then summarized by respondent type (participant vs. compañeras).

For adoption, appropriateness, sustainability, shared power, and capacity building outcomes, the semi-structured interviews with CBO administrators, recruiters, and compañeras were analyzed using the similar methods [29] using Dedoose software [30] as described for the acceptability-related semi-structured interview data. Semi-structured interview responses were triangulated iteratively across respondent types [31]. Analysis started with one author (JS-O) coding interview transcripts to identify themes and constructs that aligned with Proctor’s [19] implementation outcome definitions and equity outcomes described in the Conceptual Model for Evaluating Equity [20], using Dedoose to create an initial structured outcome codebook organized by outcome. The structured outcome codebook was then used to code two transcripts over two rounds of iterative coding by two coders independently. Any outcome codebook modifications were discussed with JS-O. Any remaining transcripts were coded using the modified outcome codebook. Codes by outcome were then reviewed by all coders and any discrepancies were discussed until consensus was reached. Once consensus was reached, the most relevant quotes were highlighted and extracted from the transcripts, and the quotes were translated into English, if in Spanish, for reporting purposes.

## Results

We conducted semi-structured in-depth interviews with all three CBO administrators, four of five recruiters (one was unavailable), and five of six compañeras (one was unavailable), and nine of 10 participants sampled (one was unavailable). Sixty of 65 participants completed the program evaluation survey (5 were unable to be reached).

### Implementation outcome: feasibility

CBOs reached their enrollment goal of 50 women each (two enrolled 50 women and one enrolled 53 women). Across sites, 231 women were invited to participate, and 24 were ineligible. Of 207 eligible, 54 refused for a recruitment rate of 74% (207/231). We randomly assigned the final sample of 153 women to the intervention (n = 76) or control group (n = 77). Six-month retention was 92% overall (140/153), 88% for the intervention group, and 95% for the control group.

Of 76 women randomized to the intervention group, 65 (86%) completed at least 7 of 10 sessions (9% completed 1 to 6 sessions, and 5% completed no sessions). Primary reasons women did not complete ≥ 7 sessions were no longer needing a support program because they had completed active treatment, treatment side effects, and travel.

### Implementation outcome: fidelity

#### Participants’ adherence and uptake

Across sessions, participants’ knowledge (> 85% correct on seven questions) and uptake of skills mastery were high (> 85% able to correctly perform all 14 skills); Table 2. Every week except the first, participants were asked to complete a weekly goal reinforcing a skill covered that week. For weeks 2–5, participants were asked to complete a distress thermometer to measure anxiety and practice deep breathing at home. At week 2 and 3, 78% and 79% of participants completed the distress thermometer, decreasing each week thereafter, with 61% completing it by week 5. Completion of deep breathing was consistently high for weeks 2–5 (83%). Completion rates for other weekly goals ranged from 72 to 88% except that only 19% were able complete the weekly goal of talking with the person with whom she had difficulty expressing herself. The most frequently mentioned problems with completing the weekly goal, from most to least often were: too busy; side effects; forgot; unable to play the DVD; and did not know how.

**Table 2:** Compañeras assess participant’s uptake of knowledge, skills mastery, and weekly goal completion (N = 76)

Week-Module	Knowledge: Participant described…	% Yes	Skills Mastery: Participant was able to….	% Yes	Completed Weekly Goal: Participant was able to…	% Yes
1) Managing the impact of cancer	Some common reactions to a breast cancer diagnosis	99	*Not assessed*		*Not assessed*	
	The benefits of deep breathing	99				
2) Breast cancer & survivorship	What is breast cancer	92	Identify questions to ask her doctors about breast cancer	88	Complete the distress thermometer*	78
			Describe how to use a survivorship care plan	96	Practice deep breathing	89
3) Finding cancer information	*Not assessed*		Describe how she would ask her doctor questions about her cancer	92	Complete the distress thermometer	79
			Describe how to use the *Cancer Information Service*	89	Practice deep breathing	88
4) Getting support	Some ways on how she can help her family cope with cancer	93	Describe some useful skills to express her feelings/needs to others	92	Complete the distress thermometer	72
					Practice deep breathing	90
5) Managing thoughts & mood Part 1	What are unhelpful thoughts	89	*Not assessed*		Complete the distress thermometer	61
					Practice deep breathing	83
	What are helpful thoughts	89			Talk with the person she had difficulty expressing herself with	19
6) Managing thoughts & mood Part 2	*Not assessed*		Identify an unhelpful thought she had	90	Apply ‘Yes, But’ to an unhelpful thought she had	79
			Identify a helpful thought she had	90		
			Describe some skills she used to change unhelpful to helpful thoughts	86		
7) Stress management	*Not assessed*		Identify some of the new relaxation techniques learned	89	Think of her own coping statements	72
					Practice one of the new relaxation techniques	83
8) Setting goals to feel better	*Not assessed*		Identify at least two activities that can help her feel better	88	Complete the goal she set to help her feel better	86
			Set achievable goals to do activities that can help her feel better	88	Complete the goal she set to be more active	88
9) Setting goals for a healthy lifestyle	Some benefits of walking or exercise	86	Identify some ways to live a healthier life	86	*Not assessed*	
10) Program recap & future goals	*Not assessed*		Identify at least two activities that can help her feel better in the future	88	*Not assessed*	
			Set clear and achievable goals to take care of herself in the future	88		

*NOTE: participants completed these same two goals for the first three sessions

#### Compañeras adherence to and quality of program delivery

Mean fidelity ratings across the 6 compañeras ranged from 3.8 (modeled skills presented) to 5.0 (used supportive/caring communication) on the 1–5 rating scale (Table 3). All ratings of the extent to which the compañeras encouraged the participant to practice each of the skills were “all” or “most” of the time, except for seeking cancer information/asking doctors questions (one compañera encouraged it “some of the time”).

**Table 3:** Compañera supervisor’s direct observation of compañeras’ fidelity to program and quality of delivery

Compañera Behavior	N = 8
In the observed session, the compañera…	Mean (SD)
Followed the manual	4.6 (0.5)
Used easy-to‐understand language to explain concepts	4.6 (0.5)
Checked for participant comprehension	4.1 (0.8)
Modeled skills presented	3.8 (1.0)
Used supportive/caring communication	5.0 (0)
Provided participant praise/feedback	4.6 (0.5)
**In the observed session, how much did the compañera encourage participant to practice…** ^**a**^	**n**
Cognitive reframing	
All of the time	3
Most of the time	1
Not applicable to session	4
Seeking cancer information/asking doctors questions	
All of the time	3
Some of the time	1
Not applicable to session	4
Good communication skills	
All of the time	3
Most of the time	1
Not applicable to session	4
Stress management skills	
All of the time	3
Most of the time	3
Not applicable to session	2
Asking others for help	
All of the time	2
Most of the time	1
Not applicable to session	5
Increasing helpful activities	
All of the time	6
Not applicable to session	2
Set goal for self-care/healthy eating	
All of the time	5
Not applicable to session	3

^**a**^ The compañera supervisor used a structured rating scale (1 = not at all to 5 = all the time) to rate each skill observed or selected “not applicable to session” if the skill was not part of the program session protocol

The compañera supervisor (direct observer) rated highly the compañeras’ ability to deliver the program as designed, and this was supported further by compañeras’ ratings of participants’ ability to learn new information, master skills, and complete most weekly goals.

### Implementation outcome: acceptability

Of 65 intervention group participants who completed ≥ 7 sessions, 60 (92%) completed the program evaluation survey.

#### Program format preferences

Regarding program timing, 55% would have preferred to have started the program when they were diagnosed, a quarter when they were undergoing treatment, and 18% after (Table 4). No one reported preferring fewer sessions, over two-thirds (72%) preferred the same number of sessions, and 28% would have preferred more sessions. Almost all (95%) preferred the program as delivered (in person) rather than by telephone or manual only; some preferred group meetings (18%).

**Table 4:** Participants and compañeras acceptability of program timing, format, length, and program quality

Participant program evaluation survey results (N = 60)			Summary of semi-structured interviews with participants (N = 9) and compañeras (below dotted line) (N = 5)
Survey question	Response choices	N (%)	
**Program Format**			
When would you have liked to have started the program. When…	• Diagnosed with cancer • Undergoing treatment • Treatment was done • Missing	33 (55) 15 (25) 11 (18) 1 (2)	Participants preferred program be offered earlier in breast cancer (BC) journey (when diagnosed or undergoing treatment).
			Compañeras suggested program be delivered earlier in BC journey.
The program consists of 10 sessions. Would you have preferred…	• Less sessions • The same sessions • More sessions	0 (0) 43 (72) 17 (28)	Participants were satisfied with the number of sessions received. Suggested offering more or longer sessions, and having follow-up sessions after program ends.
			Compañeras suggested making program sessions longer.
How would you have preferred to receive the program… (select all that apply)	• One-on-one meetings • On the telephone • Workbook only • Group meetings	57 (95) 0 (0) 0 (0) 11 (18)	Most participants preferred receiving individualized sessions because it was convenient and private. Suggested making a combination program (individualized and group) to be able to speak to a compañera in private and also meet other women.
			Compañeras suggested participants would benefit from meeting other women in the program.
**Program Quality**			
How would you rate the overall quality of the program	• Poor • Fair • Good • Very good • Excellent	1 (2) 5 (8) 13 (22) 41 (68)	Overall participants liked everything about the program, especially that it was in Spanish. The program helped them feel less isolated, manage depression and stress, feel more positive, enjoy life, and express their emotions.
			Compañeras liked the program since it was designed in Spanish for Latinas with BC.
How would you rate the overall quality of the DVD	• Poor • Fair • Good • Very good • Excellent • No DVD player	1 (2) 1 (2) 8 (13) 19 (32) 27 (45) 4 (7)	Most participants liked the videos (saw videos on the compañera’s tablet); some said that they did not own a DVD player.
			Compañeras commented that many participants did not have a DVD player.
How would you rate the skills of your compañera	• Poor/Fair • Good • Very good • Excellent	2 (3) 16 (27) 42 (70)	Participants spoke highly of compañeras’ ability to listen, provide clear explanations, establish rapport (e.g., patient, trustworthy, supportive); personal contact with a BC survivor helped them feel understood, normalized their BC, and gave them hope. Suggested they be allowed to remain in contact with compañera after the program ends (many missed their contact with the compañera).
			*Not applicable for compañeras*
**Program Usefulness**			
How much did the program help you cope with your BC	• Not at all/A little bit • Somewhat • Quite a bit • Very much	1 (2) 23 (38) 36 (60)	The program helped participants normalize their BC, how to manage side effects and stress, and understand what to expect in the future. It also helped them learn how to communicate with family about their BC, especially children. It was also helpful to have someone to talk to about their BC.
			Compañeras said they noticed positive changes in participants, as well as improved family relationships and being more relaxed.
How useful was the section on BC information and BC videos	• Not at all useful • A little useful • Somewhat useful • Quite useful • Very useful	2 (3) 3 (5) 12 (5) 43 (72)	The program helped participants better understand their BC, taught them about how to care for long-term/late effects of treatment, and taught them how to access more information. The BC videos usefulness was mixed. Some said they watched the BC videos with their family, while others said they would have preferred seeing the video when they were initially diagnosed.
			Compañeras talked about how participants liked learning about BC. The BC and treatment videos were useful for explaining it to participants.
How useful was the survivorship care plan (SCP)	• Not at all useful • A little useful • Somewhat useful • Quite useful • Very useful • Missing	1 (2) 5 (8) 11 (18) 23 (38) 19 (32) 1 (2)	Few participants discussed the usefulness of the SCP. Those that did, said it was useful to have information in one place.
			Compañeras suggested having participant’s SCP would be useful to tailor BC information to participant’s diagnosis.
How useful was the section on communicating with doctors	• Not at all useful • A little useful • Somewhat useful • Quite useful • Very useful	1 (2) 2 (3) 2 (3) 18 (30) 37 (62)	Participants liked how the program gave them skills to communicate with their doctors such as preparing questions in advance.
			*No comments made by compañeras*
How useful was the section on communicating with family members	• Not at all useful • A little useful • Somewhat useful • Quite useful • Very useful	1 (2) 3 (5) 22 (37) 34 (57)	Participants liked how the program taught them to improve communication with their family. Suggested including or offering the program to family and children.
			Compañeras suggested adding more information about intimacy and sexuality in the partner communication section; offering the program to family members.
How useful were the sections on managing thoughts and mood	• Not at all/A little useful • Somewhat useful • Quite useful • Very useful	3 (5) 21 (35) 36 (60)	Participants discussed how the program taught them to change negative thoughts to positive thoughts. They liked that they learned how to be more positive. A few women mentioned that their family or partner noticed a positive change in her.
			Compañeras said participants liked learning about how to cope with thoughts, but suggested adding more information as to why coping skills are important (some participants were reluctant to try).
How useful were the sections on managing stress and skills videos	• Not at all/A little useful • Somewhat useful • Quite useful • Very useful	2 (3) 22 (37) 36 (60)	Most participants discussed the usefulness of stress management tools and videos (watched on the compañera’s tablet). Skills helped them feel better, improved sleep, and reduced stress and depression. Many found deep breathing, and other skills such as visualization and progressive muscle relaxation useful.
			Compañeras discussed how participants liked the stress management skills.
How useful was the section on healthy living	• Not at all/A little useful • Somewhat useful • Quite useful • Very useful	1 (2) 18 (30) 41 (68)	Participants liked the section on self-care (e.g., live healthier, nutrition, diet), and learning that they need to continue self-care practices. Suggested expanding information on self-care after BC.
			Compañeras suggested expanding section by adding more information on nutrition and physical activity to manage stress.
How useful was the section on setting goals	• Not at all useful • A little useful • Somewhat useful • Quite useful • Very useful	1 (2) 3 (5) 21 (35) 35 (58)	Participants found setting weekly goals useful to achieve their own goals such as doing more joyful activities, asking for help, and returning to ‘normal’ after BC.
			*No comments made by compañeras*
**Program Ease of Use**			
How easy was it to understand the manual	• Not at all easy • A little easy • Somewhat easy • Quite easy • Very easy • Missing	3 (5) 3 (5) 27 (45) 26 (43) 1 (2)	Participants liked workbook’s large font, simplicity. Most did not have difficulty doing weekly activities. For those that did, they reported following workbook examples or compañera’s instructions. A few women said that the program content was repetitive.
			Compañeras said program content was repetitive in some places.
How convenient was the program	• Not at all/Slightly convenient • Fairly convenient • Very convenient	7 (12) 53 (88)	Participants thought program was convenient because compañera came to her home or they met at the community organization.
			Compañeras had challenges scheduling weekly visits, and dealing with last minute cancellations and long travel distances.
How often are you practicing now the skills you learned in the program	• Never • Rarely • Sometimes • Often	1 (1) 1 (1) 12 (20) 46 (77)	Participants reported still practicing deep breathing and cognitive reframing, especially during BC follow-up visits or when feeling stressed. Also discussed still using the workbook when they had a concern or question.
			*Not applicable for compañeras*

Qualitative results from participants and compañeras confirmed quantitative results (Table 4) with the following program improvement suggestions: (1) offering the program earlier (at diagnosis or treatment initiation), (2) offering more or longer sessions (weekly sessions lasted on average of 93 min (SD = 18.2) from program tracking form), and (3) augmenting individual sessions with 1 or 2 group sessions.

#### Overall quality of program, DVD, and compañeras

The program was rated as very good/excellent by most participants (90%). The DVD was rated as very good/excellent by 77% and poor/fair/good by 17%. Few respondents (7%) lacked a DVD player. Most respondents (97%) rated compañeras’ skills as very good/excellent. Suggestions from qualitative participant and compañera interviews included having alternative ways of playing the videos. Participants related strengths of the program as having personal contact with a breast cancer survivor.

#### Perceived program usefulness

Almost all participants (98%) rated how much the program helped them cope with breast cancer as quite a bit/very much. The highest ratings pertained to communicating with doctors (92% quite/very useful) and family members (94%), managing thoughts and mood (95%), managing stress (97%), healthy eating (98%), and setting goals (93%). Participants and compañeras related strengths of the program as stress management skills, communication skills with doctors and family, learning to cope with thoughts and mood, and goal setting. The lowest ratings were for cancer information (77%) and survivorship care planning (70%). Participants found the breast cancer treatment video less applicable to their post-treatment circumstances.

#### Suggestions for program improvement

Participants and compañeras suggested including family members. Compañeras suggested expanding content on managing thoughts and mood and healthy living and having access to participant’s survivorship care plan to tailor breast cancer information.

#### Program ease of use

Ease of understanding the manual (88% reported being quite/very easy) and program convenience (88% reported being very convenient) received high participant ratings. About 3/4 (77%) of participants reported currently practicing the skills often. From the qualitative results, participants and compañeras said the program was easy to use but found some content repetitive. Participants found the program convenient because sessions were delivered in participants’ homes or CBO sites. Compañeras identified challenges with scheduling sessions and travel distances (mileage was reimbursed). Compañeras’ mean weekly round trip travel time was 37 min (SD = 24.4) and mean round trip travel distance was 29 miles (SD = 24.5).

### Implementation outcome: adoption

Administrators had been employed at their organizations for > 10 years. Four themes were identified regarding administrators’ decision to adopt *NA-II* (Table 5). Administrators described that the program *aligned with their CBO’s priority population, mission and/or service model*. CBOs were well established in their community, providing services to rural, medically underserved, primarily Spanish-speaking Latino populations. Administrators decided to adopt the program because it filled a community need.

**Table 5:** Program adoption and appropriateness from perspectives of CBO administrators, recruiters, and compañeras

Theme and sub-themes	Illustrative Quotes
**Implementation Outcome: Adoption**	
**Aligns with CBO’s priority population**	“We are a very poor community, 85% of the people we’re serving are Latinos. It’s a very disadvantaged community: we’re medically underserved, we’re rural, and so their needs are very complex.”–CBO administrator
**Aligns with CBO’s mission and/or service model**	“Well, it made it attractive because it’s the type of work that we’re already doing. We provide a lot of health education to the community at no cost to them, working with promotoras and we are doing that work in English and Spanish. I knew there was the need in our area.“–CBO administrator “We are a community-based organization that focuses in approaching the patient, which makes us a very unique and strong model. We have strong social capital in our community. So, when we have the opportunity to work with our patients, work with the medical system, locally and out of county, and work with the community at large, I think we just have a very good reputation for being supportive and able to help in whatever capacity we’re functioning.”–CBO administrator
**CBO’s existing resources**	
• CBO’s internal resources	“We are a hospital and have a data analyst that would send me the list of women with certain indicators to make sure that they could participate in the program.”–CBO administrator “We’re very good at integrating activities and commitments ourselves; the team is very versatile and adaptable. So, if someone is unable to be available, the other staff easily steps in and provides support as they can. They are social workers, they’re all CITI-trained, they have a very high set of expertise that I trust very well.”–CBO administrator
• CBO’s existing relationships with external organizations	“One of my coworkers, who was also one of the recruiters, has established incredible connections in the community with medical teams and she took advantage of it in trying to recruit people. We have good connections with nurse navigators and social workers, and they were helpful in referring women to us. I think those connections and our history of being involved in the community were certainly a benefit.“–CBO administrator “We have memorandums of understanding with two oncology clinics and a radiation clinic. Since we’re not clinic-based or hospital-based those clinics are key, which gave us the opportunity to immerse ourselves in the clinic.”–CBO administrator
**Funding agency’s priorities**	“We’re familiar with the funder, and the research that’s produced, the translational research that they offer is very good. So, we wanted to be a part of it.”–CBO administrator “I always have a hard time when a project only serves one particular cancer. We have women that have other cancers. It’s just that narrow focus is hard. And I know that’s often where the funding is, but nevertheless, I think it’s a disservice to so many women in rural areas.“–CBO administrator
**Implementation Outcome: Appropriateness**	
**Program model and research process**	
• Program fit	“This program fit in really well because a lot of our population are used to working with community health workers and we had done some home visits in the past. And also, because we’re rural it’s kind of hard to get the women together to do a group. So, I think doing the one-on-one and being able to do the program in all the different areas that we serve.“–CBO administrator “I’m speaking about the curriculum. It was a great resource for women who are breast cancer survivors and the fact that it’s in Spanish and it’s delivered by a compañera, somebody who has gone through that problem themselves.”–CBO administrator “At the beginning when we talked about this program, it seemed interesting to me because it was going to be in the Spanish language and for the first time it was going to focus on Latina women.”–Compañera
• Input on program	“Before we even started, we were given the opportunity to give feedback on the program. I think it was even expanded based on some feedback. It got tailored…we anticipated what those needs were going to be.”–CBO administrator “There has never been a program that was supportive to our women with breast cancer. We’ve never had this type of support, peer support, in Spanish before, utilizing the tools that they did, that they used with the patients in their home, it was most appropriate.“–CBO administrator
• Input on research methods	“[The academic team] took our input about the survey. They made it shorter. And they changed some of the hard words on the consent form.”–Community recruiter
• Flexibility with locale	“If we had space available, they could use it for the [enrollment] interview or compañeras could meet with participants. In some instances, the participants were more comfortable coming to our office because it’s private and they’re able to focus without any distractions at home, no phone or family.”–CBO administrator “Normally I would see women in their home because they did not have transportation.”–Community recruiter
**Staffing**	
• Hire community members	“You know, the fact that we would be able to provide some type of part-time employment to a few community members. We were able to hire three *per diem* staff through this grant, anytime we can do that, that’s amazing.”–CBO administrator
• Overseeing staff	“I had not previously known either [compañera], and one of them was wonderful, and the other one was not very prompt or very good at communicating. I think she let [the academic partner] know that she went out of town, but she didn’t let me know, and I would be calling her, and then I would find out that she was out of town.”–CBO administrator “I personally do a good job with my volunteers here in the agency, but somehow I had a blind spot, and I didn’t translate that to how valuable it would have been to check-in with program staff. The program allowed my employer to add a few hours a month to my schedule. But sometimes I felt like I wasn’t able to give as much time as I ideally would have liked to.“–CBO administrator
• Staff turnover	“We had to pause our recruitment because we lost a compañera. We found a new one, but we had a hard time assigning women to her because she was only available in the late afternoon, so we had to let her go. The last [compañera] worked out.”–CBO administrator
**Implementation Outcome: Sustainability**	
**Need for new funding sources**	“Funding, that’s always the question when we think about sustaining the program in the future. Actually, last week, I had a conversation with our foundation director, and she asked about any projects that we wanted to do and needed funding for. So, I mentioned Nuevo Amanecer and I think we found a source that we’re gonna apply to. I’m gonna ask [the academic partner] to provide me with some preliminary data that I can use on the application.”–CBO administrator “We are a Medicare provider, and we are preparing to become a Medicaid provider. We can bill for psychotherapeutic support. That’s within our capacity and within our designation. So, this skillset from Nuevo Amanecer is billable through us. We want to expand this skillset, doesn’t matter the type of cancer, that is where the strength for perpetuating sustainability is for us. It would be through our capacity to bill Medicare, Medicaid, and private insurance.”–CBO administrator “At this point we don’t have funding to pay compañeras to do the one-on-one ten-week program. We might be able to do it in a group setting. We’re hoping to continue to take advantage of and utilize the program because it’s extremely valuable, but at this point we don’t have any funding ideas.”–CBO administrator
**Incorporate aspects of program into current CBO services**	“We’ve talked about perhaps one of the compañeras delivering some levels of the program to our support groups, you know, our Latina support groups or something like that.”–CBO administrator “Our intention is to continue this very good, structured model, but we’ll integrate it within our own capacity. Maybe not in its purest form and we are limited in home visits. However, we are able to integrate the majority of the coping skills and the program within our agency in either individual or group form.”–CBO administrator

Administrators highlighted how they capitalized on their *existing CBO resources* (with two sub-themes). Sub-themes included availability of CBO *internal resources* (e.g., staff, electronic records, physical space, knowledge of population) for program implementation, and *pre-existing relationships with external organizations* (e.g., established connections with medical staff, community resources) to obtain program referrals or additional resources (e.g., medical, food, cash assistance).

### Implementation outcome: appropriateness

We identified two overarching themes (program model and research process, staffing) on contextual factors that supported/hindered appropriateness (Table 5). Respondents discussed how the *program fit* their community setting because it utilized community health workers, home visits, and one-on-one delivery given common barriers in rural areas (e.g., travel distance, large geographic service area). Administrators and compañeras stressed that elicitation and incorporation of their *input on program* and training materials prior to implementation was a major strength.

Recruiters commented on how the academic partner made adaptations to the research methods (e.g., simplified wording, shortened survey) based on their *input on research methods* so they were more appropriate and easier to use. Administrators and recruiters discussed that having the *flexibility with locale* to conduct program sessions or enrollment at the CBO office or women’s homes made it practical.

Administrators discussed how *staffing*, with three sub-themes (hiring community members, overseeing staff, staff turnover), affected appropriateness for their setting. Administrators talked positively about their ability to *hire community members* to deliver the program or enroll women. They also expressed challenges *overseeing staff*, e.g., role confusion or lack of communication.

Lastly, *staff turnover* was a limiting factor. One organization lost one of their compañeras and had to pause recruitment until the position was filled. Another organization had difficulty identifying a suitable, bilingual recruiter.

### Implementation outcome: sustainability

Administrators identified two sustainability related themes (Table 5). All administrators discussed the *need for new funding sources* to sustain the program as delivered. Two administrators talked about potential funding sources (foundation grant, billing Medicaid). All administrators discussed how they would *incorporate aspects of program into current CBO services* such as delivering skills training within their support groups. They discussed keeping the program but altering the delivery mode to a group setting or individual sessions at the CBO (no home visits).

### Equity outcome: shared power

We identified five themes regarding shared power (Table 6). Administrators spoke of the *partnership structural dynamics* (e.g., funding allocation, memorandums of understanding) that supported program implementation and indirect staff (e.g., administrative assistant, data analyst). All three administrators spoke highly of the *partnership collaboration* and the high-quality communication that provided *opportunity for information exchange* between CBO and academic partners. They valued the monthly conference calls to address issues, troubleshoot, and brainstorm solutions. There was resounding agreement among the administrators about the professionalism of the academic and lead community partner and the sense of *mutual respect*. Administrators spoke about the *academic team’s reputation working with community* as a strength of the partnership. One administrator said that they joined the partnership because a colleague from another organization (not involved in the project) recommended the principal investigators and program.

**Table 6:** CBO administrators, recruiters, and compañeras perceptions of shared power and capacity building

Theme and sub-themes	Illustrative Quotes
**Equity Outcome: Shared Power**	
**Partnership structural dynamics**	“Funding is always an issue, it’s always helpful. We were grateful that the compañeras were able to be paid, and as an organization to be given some financial support. In any type of research project, I think that community partner must be considered, to be given some type of financial support. And we did appreciate the funding that was afforded to us. More would’ve been great but understood the tight nature of the funding constraints.”–CBO administrator
**Partnership collaboration**	“I appreciated the fact that they were very team player, team oriented. So, it was always us, ‘We’re doing this project.’ It always involved everybody else. It wasn’t just, ‘Oh, you know, it’s our project and you guys are just helping us.’ Every step of the way, it was very ‘our project’.“–CBO administrator “I think the research staff were incredibly supportive; they were very respectful and engaging and collaborative at every step of the way.”–CBO administrator
**Opportunity for information exchange**	“We were always communicating. We had the monthly conference calls that were really helpful for the staff. Any issue that came up, we were able to resolve them. Having experience with other projects, their communication was excellent compared to other ones that I worked with.”–CBO administrator
**Mutual respect**	“[The academic team] are very professional. They’re very respectful of who they are helping, the individual, the participant, and very respectful to us as organizations. And I appreciated that, because there was an awareness of the impact to our very busy space, and yet, our commitment to making sure that things were rolling out well.”–CBO administrator
**Academic team’s reputation working with community**	“Working with [academic principal investigator] team - I’ve worked with many researchers, and she’s very professional, she’s very sensitive to the community, sensitive to the needs of the organization, and she’s a professional. And so, for us, it’s very important who we partner with.“–CBO administrator “I think the study’s structure itself and the caliber of research that was occurring is very refreshing. We’ve participated in a lot of different studies; most were excellent, a couple were not. And so, I’m pretty selective, now, who I partner with, and so working with [academic partner principal investigator] is a great research partner.”–CBO administrator
**Equity Outcome: Capacity Building**	
**Training and technical support**	
• Training	“I think the training you gave us is terrific. Putting it into practice at first one feels nervous. But once you start, well, you learn along with [participants]. But I think the training needs to be extended a little more in–in some areas that would prepare us a little more.”–Compañera “Well, [the compañera supervisor] did the training for the compañeras and that went over really well.”–CBO administrator “One of my recruiters expressed the desire to have been included in the compañera training…so that she had a broader, more comprehensive understanding of what was going to be offered to the women.”–CBO administrator
• Providing ongoing technical support	“[The compañera supervisor] came down and did a couple of site visits and reviewed with the compañera.”–CBO administrator “[The academic partner] even came down and went to like six or seven doctors’ offices with one of the recruiters. They went together and did that outreach to connect with doctor’s offices, you know, that was wonderfully supportive.“–CBO administrator “[The academic team] was always there to support us, they answered us quickly, they cleared up our doubts.”–Recruiter “The only thing that I can think of – or if they offered this or not – talking to women can be very emotionally draining, and so sometimes, the compañeras needed to talk to somebody just to kind of process those feelings and emotions so maybe offer some additional support for them. But I know that [compañera supervisor] was available to them if they needed to do that.”–CBO administrator
• Role modeling	“At first for me [the program] was difficult, but with the help of [the other compañera] I learned a lot by observing her.”–Compañera “Also, my colleagues, the other recruiters from the other agencies, shared their [recruitment] experiences, those helped too.”–Recruiter
**Individual-level capacity**	
• Compañeras enhanced capacity to deliver program	“I thought that perhaps I had not learned anything [in the training] and maybe needed more training, but when I had to start, I turned to the manual, and that’s when I started to realize that I could do it. Also talking to [participants] about our own experience and about the experience they are going through, it was very easy for me. It was not difficult for me.”–Compañera
• Compañeras self-application of program skills	“I feel that I learned a lot because I think that it helps in all aspects of life, not only with the cancer. I think that it would help us in everything. There are times that with the simple fact of work, one is very negative, one is very stressed. So, I feel that this helped me in all areas of life, not only with cancer.”–Compañera
• Recruiters increased research skills capacity	“I also think it was successful because it expanded into the saliva and hair samples for the study, which historically, Latinas are pretty aversive or they’re a little guarded when it comes to participating in studies. So, the beautiful way that the recruiters were able to explain the whole process in a trusting way was really beneficial.”–CBO administrator “I only wanted to say, at school when I was in my master’s degree we talked about research. And with this program, I saw it in action – what a control group and intervention group are, the surveys, and how it all comes to life instead of reading it in a book.”–Recruiter
• Recruiters’ increased knowledge	“For me working at the agency with women, I see how they come, anxious or sad because of their diagnosis. But it was a very different experience when I went to their homes to do the interview. I got to look at other aspects of her life. When I was in their houses, they talked about topics that they would not mention here. That was something that left an impression on me. I think that it helped me to grow to look beyond their diagnosis, to think about everything else that people are going through or have gone through.”–Recruiter “I think that for me, the questionnaire opened up my perspective. For example, asking the questions about if she is sexually satisfied or questions like depression, anxiety, I could see how they were in those aspects, and I could suggest a gynecologist or something else. I think that opened up my perspective a little bit.”–Recruiter
**CBO-level capacity**	
• Enhanced CBO program capacity	“Well, I guess it increased our capacity because we were all trained on this new curriculum on this new program that we’ll be able to offer the community in the future.”–CBO administrator “When we are given something to help the people we serve, we will maximize it. This skillset of coping skills can be expanded we can utilize it with men, we can utilize it with women, with any cancer, in any stage. When we participate in research, it is an opportunity for my agency and for my staff for capacity-building. We don’t have a budget for staff development. And so, we adapt to the needs of the research project with our own skillset, but we expand our skillset.” –CBO administrator
• Enhanced CBO research skills capacity	“I learned a lot. Where do I start? So, I learned, of course, about the IRB process and why that’s important. I learned about what it means to be part of a research study, how to keep everything ethical as possible.”–CBO administrator “We would be receptive to another study. I’m thankful that we had this experience because it prepared us to expand to other research projects that we are currently engaged in. Research is a very important element within our organization, as is the direct patient experience, and the capacity-building of my staffing, as well as the organization overall. We just applied for our first grant as Co-PI [with the same funder] and it’s my first experience as a Co-PI.”–CBO administrator
• Enhanced CBO’s reputation	“It helped bring awareness to the community, it helped us to better articulate and market what we’re doing as an organization –helps towards the sustainability of our organization and our work.“–CBO administrator “The other thing, too, that we didn’t anticipate, is the hospital decided to do an open house and so, they sent a press release. They included Nuevo Amanecer, that was really interesting to the media. We got a call from *Univision* and I did an interview about the project. So, that was something a little bit unexpected, and I think also, it makes our organization look in a positive light for the community, because we are involved in these types of studies.”–CBO administrator

### Equity outcome: capacity building

We identified three overarching themes related to capacity building, training/ongoing technical support, individual-level capacity, and CBO-level capacity (Table 6). With respect to *training and technical support*, three sub-themes emerged (training, providing ongoing technical support, and role modeling). Compañeras and recruiters spoke highly of the *training* provided and that they felt prepared for their duties. Compañeras would have liked training on additional breast cancer related content (e.g., intimacy, sexuality, nutrition). An administrator suggested that compañeras and recruiters be cross trained on each other’s roles to address turnover. Administrators at times were unsure how to best support recruiters and compañeras given the roles of the academic and lead community partner.

Informants reported that a key determinant of success was *provision of ongoing technical support* to compañeras and recruiters by the academic team and lead supervisor. Compañeras, appreciated receiving positive reinforcement and feedback on program delivery via fidelity checks. However, they mentioned that fidelity check visits were difficult to coordinate given that they required alignment across supervisor, compañera, and participant schedules. Conducting fidelity checks required the supervisor to travel from 100 to 600 miles to study sites. In addition to the monthly meetings of all staff, there was a sentiment that compañeras needed more support to process their interactions with participants.

Recruiters discussed that the academic team was readily accessible to answer questions. Two administrators discussed how well the academic team supported their recruiters. Another administrator talked about how the academic team assisted them in developing institutional review board approved procedures to recruit potential participants using hospital records, providing template letters and post cards. Lastly, compañeras and recruiters spoke highly of the value that *role modeling* had on enhancing their program delivery or recruitment capacity.

Compañeras reported their individual-level *enhanced capacity to deliver the NA-II program* and their *self-application of program skills* to their personal lives. Several compañeras wished they had had access to this type of program when they were undergoing their diagnosis. Recruiters reported *increased research skills capacity* and *increased knowledge* of the psychosocial impact of breast cancer.

At the CBO-level, administrators talked about *enhancing their CBO’s program capacity*. They saw the cognitive behavioral coping skills as being transferable to individuals with other cancer types/other genders. Administrators indicated that because the organization had limited staff development funds, staff were able to obtain skills through the study that they otherwise would not have. In addition, it *enhanced their CBO’s research skills capacity* to participate in other research projects with other researchers. Lastly, administrators discussed that their participation *enhanced their CBO’s reputation* in the community through media coverage or town halls of their CBO’s participation in *NA-II*.

Distilled from the data, we summarize best practices for how investigators can equitably engage community organizations to implement behavioral interventions (Table 7). Highlights include provide compensation for community partners to engage in all phases of the research, create synergy on mission and priorities, build on community assets, build further community capacity, and provide ongoing technical assistance throughout implementation.

**Table 7:** Best practices (lessons learned) to equitably implement behavioral interventions and engage community organizations

Prior to funding
• Principal investigator should identify as a community-based researcher or health equity researcher and be familiar with related principles, frameworks, and methods. • Identify areas of synergy across academic and community partners, ensuring a match with CBO’s mission, priorities, and/or service model. • Identify funding mechanism and resources for paying community partners for their involvement; if possible, funding should not have many restrictions on priority focus areas. • Establish and maintain open communication, mutual respect, and professionalism between academic team and community organizations – through all phases.
**Post funding/Pre-implementation**
• Set up memorandum of understanding (MOU) between partners to allocate adequate funds for program implementation, specify roles, deliverables, etc. • Diversify academic team; team members should identify with focus/priority population. • Hire community members as implementors who have flexible schedules to accommodate participant preferences. • Identify CBO’s resources (i.e., internal, external) that could potentially be used for program promotion and implementation. • Provide CBO administrators with program management skills or tools to engage with implementors and end-users. • Cross-train implementors in both outreach/recruitment and program delivery. • Academic and community partners co-create, tailor and/or adapt program content, materials, and data collection tools or forms. • Identify private space for program delivery or to conduct study interviews that is feasible for community organization and members.
**Throughout implementation/Post-implementation**
• Identify a point person that implementors can contact if there is a participant who feels unduly distressed or needs additional support (e.g., referral to other local resources). • Provide ongoing technical support throughout the implementation process for implementors. • Provide alternative learning methods for implementors (e.g., practice, role modeling, observe program delivery and provide immediate feedback). • Support CBO outreach and recruitment efforts (e.g., accompany implementors to connect with new external organizations, provide recruitment letter templates, etc.). • Identify alternate funding revenues for sustainability or key program components that can be integrated into current CBO services.

*Implementors is a broad term we use to describe those involved in the implementation process (in *NA-II* implementors refer to administrators, compañeras, and recruiters)

## Discussion

This study used an innovative, comprehensive, mixed methods approach to evaluate the implementation processes of *NA-II*, a peer delivered stress management program designed for Spanish-speaking Latinas with breast cancer in partnership with CBOs serving three rural California communities. A broad implementation evaluation framework was applied to explore various implementation process outcomes, supplemented with an equity evaluation framework that went beyond traditional implementation science outcomes.

Our choice of this broad approach was based on the innovative *NA-II* design, which was guided by the Transcreation Framework for Community-Engaged Behavioral Interventions to Reduce Health Disparities [21]. The Transcreation framework describes a 7-step process that fully engages the community in planning, delivering, and evaluating a program. It emphasizes principles of shared decision making, equal value placed on scientific and community knowledge, and building capacity to conduct future programs. The CBOs were thus engaged in program adaptation, implementation, and evaluation through monthly meetings that included all community-based staff (administrators, recruiters, and interventionists) in addition to the academic partner and lead community partner staff. The implementation evaluation thus required a matching approach that reflected these principles, the perspective of all of these individuals, and that enabled evaluation of the strengths and challenges of training community-based individuals to fill these roles.

We illustrate here how we tailored our implementation evaluation to this unique approach. Our traditional implementation evaluation framework included feasibility, fidelity, acceptability, adoption, appropriateness, and sustainability. However, we defined these in somewhat unique ways. For example, fidelity was examined in terms of participants’ uptake of program components (traditional definition) as well as in terms of community-based interventionists’ ability to deliver the program per protocol (unique definition). We selected equity outcomes to evaluate the extent to which we succeeded in sharing power and building capacity. In another example, we evaluated shared power by exploring their sense of the partnership dynamics such as governance and communication.

Implementing psychosocial interventions in rural areas face considerable challenges over and above those in urban areas. Because of the paucity of services in rural areas, strategies require extensive collaboration between institutions to augment conventional delivery systems [32]. The three CBOs (mental health service organization, Latino/a-serving cancer organization, and safety-net hospital) provided varied settings for implementing the intervention. The settings were chosen for their expertise providing services to Spanish-speaking Latinas, and because *NA-II* aligned with the CBOs’ priority populations and missions. Understanding local community contexts for implementing psychosocial interventions helps address large differences that can exist across rural communities and relative to the original program test sites [33]. Contextual factors such as funding, competing demands, organizational structure, and CBO staffing needs influenced the implementation process. Program acceptability for CBO staff was evidenced, with minimal suggestions for improvement.

Equity success was due largely to use of strategies of shared responsibility and learning and co-ownership (shared power), resulting in a co-created, co-tailored program for Spanish-speaking Latinas with breast cancer and the community and organizational context. In this study, it was important that all partnership members share responsibility and ownership of intervention and data collection processes [23] for a successful implementation [25]. Training and ongoing technical assistance were key factors. Building capacity beyond the program was imperative for CBOs. A common barrier to equitably engaging community members in implementation science is CBOs’ resource limitations, thus, compensating them for their full involvement in the research process was a pre-requisite [34]. Acquisition of research skills enabled community members to apply their new skills and knowledge to subsequent projects and enabled extension of training on the program to others within their communities [35, 36].

Challenges to community involvement typically relate to communication, inclusiveness, and trust issues, which can affect implementation [25] and CBPR success [20, 37]. By evaluating the partnership from the perspectives of CBO administrators, recruiters, and compañeras (interventionists), we were able to explore the extent to which partners were engaged in co-equal decision making. Suggested best practices include providing compensation for community partners, engaging them in co-creation and adaption of intervention materials and study procedures, designing implementation processes to build on community assets, building community capacity, and providing ongoing technical assistance. Evaluation of implementation processes using data from multiple perspectives builds the evidence-base to inform future implementation [38]. Reporting complied with TIDieR reporting standards [39] (see Additional file 1).

Successful implementation of *NA-II* was due in part to the long history of the academic and lead community partners’ use of CBPR principles to test community-based psychosocial interventions among Latina breast cancer survivors. Even though the three rural CBOs were new partners, they recognized the reputations and the prior partnership of the academic and lead community partner with other CBOs to improve psychosocial health among Latinos with cancer [16, 40–43]. This credibility contributed to intervention adoption and is hyper critical in communities that have been traditionally disenfranchised by institutionalized power structures that limit access to health-sustaining resources.

### Limitations

CBOs and staff participating in our study may not be representative of other rural organizations. Results therefore may not generalize to other culturally and linguistically diverse communities and populations. While the *NA-II* intervention reduced anxiety and improved stress management skills [18], parts of it may not have been relevant to long-term breast cancer survivors. Yet findings may have implications for addressing the psychosocial needs of long-term breast cancer survivors across the care continuum and their lifespan [44]. Sample sizes were small for each type of informant and data were self-reported, potentially introducing social desirability bias (answers that they believed would please the interviewers). Finally, greater attention to sustainability would have been helpful. Two of three CBOs had plans in place to obtain additional funding to continue *NA-II;* thus more funding to provide technical assistance to achieve these plans would have been extremely helpful.

## Conclusions

Applying an equity-focused approach to co-creation, implementation, and evaluation of *Nuevo Amanecer-II* offered the opportunity for individual and organizational capacity building, an equitable partnership, and an acceptable and effective psychosocial intervention designed for a vulnerable population. The inclusion of both implementation and equity outcomes from multiple community perspectives offers a comprehensive evaluation to better inform community implementation of peer-based programs designed to address populations and settings that have experienced limited access to health preserving resources.

### Electronic supplementary material

Below is the link to the electronic supplementary material.

Supplementary Material 1

## Data Availability

The datasets during and/or analyzed during the current study available from the corresponding author on reasonable request.
